# Assessment of Phenological Dynamics of Different Vegetation Types and Their Environmental Drivers with Near-Surface Remote Sensing: A Case Study on the Loess Plateau of China

**DOI:** 10.3390/plants13131826

**Published:** 2024-07-03

**Authors:** Fengnian Guo, Dengfeng Liu, Shuhong Mo, Qiang Li, Jingjing Meng, Qiang Huang

**Affiliations:** 1 State Key Laboratory of Eco-Hydraulics in Northwest Arid Region of China, School of Water Resources and Hydropower, Xi’an University of Technology, Xi’an 710048, China; guofn@sehemodel.club (F.G.); moshuhong@xaut.edu.cn (S.M.); mengjingj@xaut.edu.cn (J.M.); wresh@mail.xaut.edu.cn (Q.H.); 2 Center for Ecological Forecasting and Global Change, College of Forestry, Northwest A&F University, Yangling 712100, China; qiang.li@nwafu.edu.cn

**Keywords:** phenology, PhenoCam, Chinese Loess Plateau, generalized additive model

## Abstract

Plant phenology is an important indicator of the impact of climate change on ecosystems. We have continuously monitored vegetation phenology using near-surface remote sensing, i.e., the PhenoCam in a gully region of the Loess Plateau of China from March 2020 to November 2022. In each image, three regions of interest (ROIs) were selected to represent different types of vegetation (scrub, arbor, and grassland), and five vegetation indexes were calculated within each ROI. The results showed that the green chromatic coordinate (GCC), excess green index (ExG), and vegetation contrast index (VCI) all well-captured seasonal changes in vegetation greenness. The PhenoCam captured seasonal trajectories of different vegetation that reflect differences in vegetation growth. Such differences may be influenced by external abiotic environmental factors. We analyzed the nonlinear response of the GCC series to environmental variables with the generalized additive model (GAM). Our results suggested that soil temperature was an important driver affecting plant phenology in the Loess gully region, especially the scrub showed a significant nonlinear response to soil temperature change. Since in situ phenology monitoring experiments of the small-scale on the Loess Plateau are still relatively rare, our work provides a reference for further understanding of vegetation phenological variations and ecosystem functions on the Loess Plateau.

## 1. Introduction

Plant phenology is the growth rhythm formed by plants adapting to environmental change, usually including germination, flowering, defoliation, and other natural phenomena [1,2]. In the context of climate change, phenological variation may alter the carbon budget and productivity of ecosystems, and affect pollinators and crop yields [3]. Therefore, a clearer understanding of the phenological dynamics of different ecosystems will not only enable a better interpretation of the response process of vegetation to climate change, but also obtain essential information on the energy exchange between the biosphere and the atmosphere, which is of great significance for agricultural production and ecological conservation in the region [4,5].

However, accurately obtaining phenological information on vegetation is challenging. Traditional research methods are founded on ground-based observation, relying on direct human observations of phenological events, and these studies are usually carried out on a limited number of individuals at specific sites [6]. The large number of ground-based observations often involve different observers and methods, which makes the interchange and integration of data from different regions difficult, thereby generating statistical noise caused by observational inconsistencies [7,8]. Despite these shortcomings, ground-based phenological observations can still accurately record the timing of phenological events for specific species [8], providing reference and comparison for remote sensing phenological studies. Over the past decades, in order to observe vegetation phenology on a larger spatial scale, satellite remote sensing has emerged as a major tool [9]. Many studies have used both NASA EOS Moderate Resolution Imaging Spectroradiometer (MODIS) [10,11], and ESA Sentinel-2 missions [12,13]. In addition to the commonly applied optical vegetation indices (e.g., NDVI, EVI), some new remote sensing metrics have been employed in phenology monitoring, such as solar-induced chlorophyll fluorescence (SIF) [14] and microwave vegetation optical depth (VOD) [15]. Compared with the traditional optical vegetation index, VOD is more suitable for phenology monitoring in subtropical or tropical cloudy regions, while SIF demonstrates its value in phenology observation of arid ecosystems or farmland ecosystems [16]. However, it should be noted that poor environmental conditions (e.g., thick cloud cover) or BRDF (bidirectional reflectance distribution function) effects can reduce the accuracy of satellite remote sensing [8,16]. Also, low spatial resolution and revisit frequency make it difficult to obtain accurate phenological phases, which is more serious in man-made forests in urban areas [17]. To compensate deficiencies of the above methods on phenological observations, near-surface remote sensing approaches based on the PhenoCam have been extensively used.

In contrast to satellite remote sensing and artificial observations, PhenoCam records time-lapse photographs of plant canopies or communities using digital cameras mounted on flux towers or masts and extracts quantitative data on vegetation color in the red, green, and blue wavebands from the images, which are then transformed to commonly used vegetation indices [18,19]. Metrics derived from PhenoCam images refine the link between satellite and field perspectives, provide higher temporal resolution, expand the spatial footprint of field observations, effectively reduce the impact of atmospheric and mixed pixels, and decrease the cost of observations, better reflecting differences in phenology of different vegetation types at sub-pixel scales [16,20,21]. This method has been applied in forest, grassland, and farmland ecosystems for monitoring phenology as well as biomass assessment due to its excellent practicality and convenience [22,23,24]. In addition to these, PhenoCam is more broadly used for satellite remote sensing product assessment as well as water and carbon fluxes interpretation [19]. In North American temperate and boreal forests, Melaas, et al. [25] evaluated the ability of Landsat phenology algorithms to reconstruct a long time-series of the start and end of the growing season for vegetation, and explored the agreement between traditional and Landsat-based phenological observations. Another study in grassland communities from China assessed the relationship between the PhenoCam-derived greenness index and carbon fluxes of grasslands, with significant correlations between them demonstrating the potential of PhenoCam for monitoring canopy dynamics in semiarid grasslands [26]. Several other studies have used digital images to investigate the relationship between canopy greenness and variation of trees’ water fluxes. For example, after analyzing the relationship between green chromatic coordinate (GCC) and sap flow as well as canopy conductance in a Mediterranean tree-grass ecosystem, Luo, et al. [27] suggested that the combination of environmental drivers and greenness can well explain sap flow and canopy conductance. Overall, the rapid development of PhenoCam-based phenological observation methods over the past decade or so has contributed greatly to the understanding of plant phenology in different ecosystems, despite the problems of vulnerability to weather disturbances and missing data due to power supply failures.

The timing of plant phenology events is influenced by different biological and environmental factors [8]. Temperature is usually considered to be the primary factor controlling vegetation phenology [28]. In the context of global climate warming, seasonal biological phenomena which depend on accumulated temperature may be impacted [29], such as earlier leaf unfolding and delayed leaf fall [30]. The study by Menzel, et al. [31] in Europe found a longer growing season for plant phenology, which is also supported by the research of Kolárová, et al. [32]. In addition to these, some studies have suggested that spring phenology is more responsive to daytime warming than to nighttime warming, and that the influence of such asymmetric warming effects should be taken into account in modelling [33]. Nutrient and water availability is another important controlling factor [8]. An experimental study on temperate trees found that the interaction between source and sink processes in regulating the end of the seasonal vegetation cycle is influenced by light, water, and nutrient availability [34]. Several recent studies have suggested that autumn precipitation has extensive influence on interannual variation in alpine grassland autumn phenology [35], and that significant interaction between precipitation and temperature also affects vegetation phenology [36]. Nutrients can also affect plant phenology, with nitrogen addition potentially delaying phenology time and shortening the duration of phenology [37]. Some other critical factors that control changes in plant phenology include photoperiod, winter chilling, resource limitation, as well as interactions among different phenological events [8,38]. These drivers, which are closely linked to plant phenological variation, are important for understanding future phenological changes, and additional experiments in different regions will contribute to understanding how these key mechanisms operate.

In this study, we investigated the seasonal patterns of leaf phenology for different vegetation types (arbor forest, scrub, grassland) in the gully area of the Loess Plateau of China with a PhenoCam mounted on a flux tower in the area. Specifically, we aimed to (1) investigate the characteristics of phenological dynamics in three typical vegetation types in the region, (2) compare the discrepancies in seasonal patterns among different species, and (3) analyze the driving factors affecting plant leaf greenness variations. There are few PhenoCam-based phenological studies in the Loess Plateau, therefore, exploring plant leaf phenology at local fine spatial and temporal scales will help to improve our understanding of interspecific phenological differences in the region.

## 2. Materials and Methods

### 2.1. Site Description

**Figure 1:**
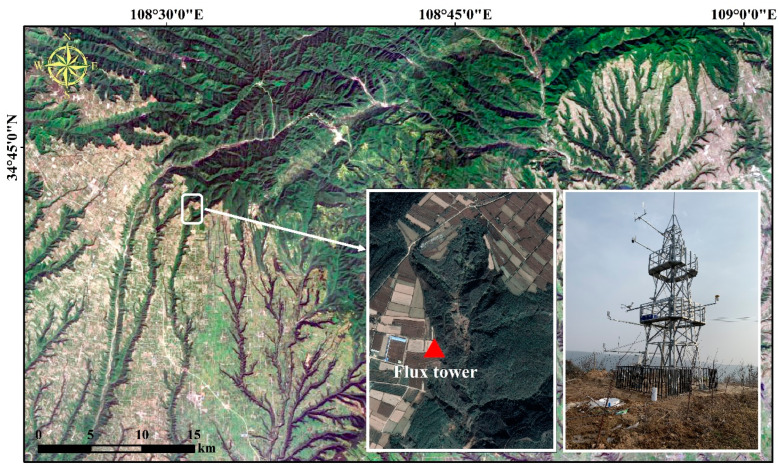
The study area located at Chunhua County, Shaanxi Province, China, on the southern Loess Plateau. The red triangle represents the position of the flux tower.

The flux tower of this study was located at Shiliyuan Town, Chunhua County, Shaanxi Province, China, where the terrain is dominated by loess gullies, and the station site is at an altitude of 1460 m (Figure 1). A continental monsoon climate exists in the study area, with an annual average precipitation of about 600 mm, and the main land use types include farmlands and vegetations. The gully area to the east of flux tower was the region we observed with PhenoCam, where *Robinia pseudoacacia*, *Rosa xanthina*, and *Artemisia eriopoda* are the primary plants that grow here. In 2019, we mounted the eddy covariance systems, PhenoCam, radiation sensors, and some meteorological observations on the flux tower, which began operating in 2020. For more details on mounting the equipment, please refer to the study by Guo, et al. [39]. During the observation period, to ensure the raw data accuracy, the flux tower was not surrounded by obstacles prone to obscuration and the eddy covariance systems were calibrated once a year.

### 2.2. Data Acquisition

A PhenoCam (NetCam SC web camera, StarDot Technologies, Buena Park, CA, USA) was mounted 5 m above the ground on the flux tower, with the lens facing northeast, which was used to capture RGB images of plants and saved them in JPG format with a resolution of 2592 × 1944 pixels. We set the camera to record at 10:00 a.m., 13:00 p.m., and 16:00 p.m., therefore, ideally, we would get three images per day; however, in reality, power supply failure or something else would cause some of the images to be missing. In this study, the images acquired at 13:00 every day were used preferentially. In addition to this, the images that were heavily affected by rain or fog were visually eliminated one by one to ensure the clarity of images. During the study period from 23 March 2020 to 30 November 2022, 932 days of images (one per day) were retained after screening for subsequent analysis. In addition to the images, daily meteorological and environmental factors data were also collected during the same period. Evapotranspiration (ET) was observed by the eddy covariance systems, which consisted of an infrared gas analyzer (Li-7500A, LI-COR, Inc., Lincoln, NE, USA) and a three-dimensional sonic anemometer (CSAT3, Campbell Scientific, Inc., Logan, UT, USA). Net radiation (Rn) and sunshine hours (Sunh) were derived from a four-component radiation sensor and a sunshine-hour sensor, respectively. Air temperature (Ta) and relative humidity (RH) were monitored by temperature and humidity sensors. We buried six soil sensors at 20 cm, 40 cm, 80 cm, 120 cm, 160 cm, and 200 cm below the surface for observing volumetric soil water content (SWC) and temperature (Ts) at different depths. In this study, we further classified them into three different depth layers: upper (soil depth less than 60 cm), medium (soil depth between 60 cm and 140 cm), and deep (soil depth between 140 cm and 200 cm). Then, for each depth layer, we calculated the mean of daily soil moisture. Precipitation (P) and wind speed (U) were measured by a rain gauge and wind speed sensor, respectively. Vapor pressure deficit (VPD) and atmospheric pressure also fell under the category of observations. These variables were transmitted over the wireless network and aggregated into daily data for subsequent analysis.

### 2.3. Image Processing and Vegetation Indexes Calculation

**Figure 2:**
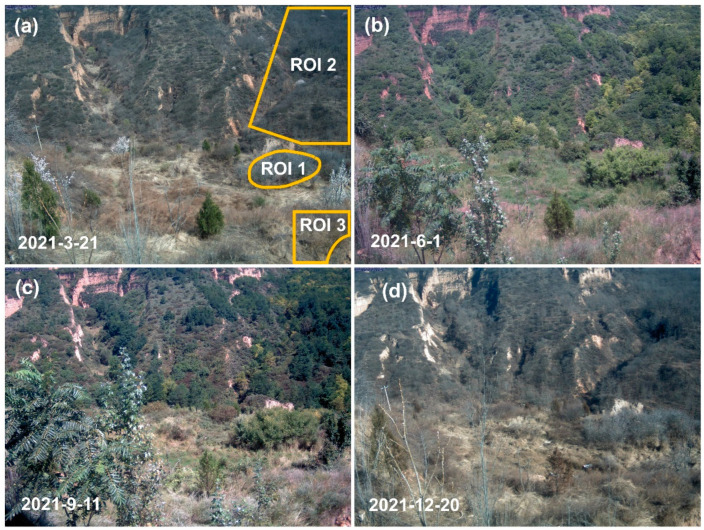
As an example, four images from different dates in 2021, the fields contained in yellow lines represent the region of interest (ROI). The vegetation types within ROI 1, ROI 2, and ROI 3 are scrub, arbor, and grassland, respectively.

In order to facilitate the extraction of information from the images, we defined the region of interest (ROI) in the screened images. In this study, three regions of interest were selected in each image (Figure 2). The vegetation type within ROI1 was scrub, while within ROI 2 and ROI 3 were arbor and grassland, respectively. Three ROIs in all images were processed on the PhenoCam GUI to extract the red, green, and blue digital numbers (DNs) from each ROI. For more details on the PhenoCam GUI, please refer to https://phenocam.nau.edu/webcam/tools/, accessed on 19 December 2023. For each ROI, we calculated five vegetation indexes separately to assess differences in seasonal patterns of vegetation described by the different indexes (Table 1). The green chromatic coordinate (GCC) could be used to characterize the seasonal trajectory of vegetation activity, which has been extensively used for phenological analysis based on PhenoCam images. The vegetation contrast index (VCI) is a nonlinear transform of the GCC, which results in a higher dynamic range for the VCI relative to the GCC [40]. Another index, excess green index (ExG), presents advantages in distinguishing between green plants and soil background [22]. The visible atmospherically resistant vegetation index (VARI) has been proven as a good indicator of the relative content of anthocyanin, and VARI can be used to detect vegetation phenological changes in autumn [41]. There are also many applications for red chromatic coordinate (RCC), which were suitable for tracking autumnal changes of foliage senescence [41]. These indexes were applied to quantify the canopy dynamics of different vegetation types and thereby compared the discrepancies between them.

### 2.4. Extraction of Phenological Metrics

Before extracting vegetation phenological metrics, it is usually required to fit or filter the data to mitigate the effects of noise within data [43]. In this study, we smoothed the time-series using the “LOESS” algorithm from the DATimeS toolbox. For details on the DATimeS toolbox, please refer to the study by Belda, et al. [44]. After smoothing the vegetation index series, the DATimeS toolbox was also used to extract phenological metrics. The start of season (SOS) was defined as the first day when the smoothed time-series increased to 20% of the amplitude of the current year. The end of season (EOS) was defined as the last day when the smoothed time-series decreased to 20% of the amplitude of the current year. The difference between SOS and EOS represents the length of growing season (LOS).

### 2.5. Statistical Analyses

Vegetation phenology may generate complex response relationships with environmental factors, and such relationships are usually non-linear [45,46]. To investigate the relationship between phenological dynamics of different vegetation types and environmental factors in the study area, we fitted a generalized additive model (GAM) using vegetation index time series as the dependent variable and the environmental data as explanatory variables. GAM is a semi-parametric regression model extended from the generalized linear model and the additive model [47], which can flexibly express the linear and nonlinear relationship between a dependent variable and explanatory variables [48]. Consequently, GAM has been widely used in the field of ecology [49]. The generalized additive model takes the following form:

(1) $$g\left(\mu \right)=\alpha +\sum_{i=1}^{n}s_{i}(X_{i})$$

where g() is the link function, μ is the expectation of response variable, α is an intercept term, $s_{i}()$ is a smooth function, and $X_{i}$ is the explanatory variables.

The GAM was fitted in R using the *mgcv* package in this study [50]. We used daily GCC of different vegetation types during the study period as the response variables and environmental factors (Rn, Sunh, U, Ta, RH, VPD, Ts, SWCu, SWCm, and SWCd) as the explanatory variables, and constructed models using thin plate regression splines function, thereby evaluating the relationships between environmental factors and plant greenness variations. SWCu, SWCm, and SWCd represent the upper soil water content, medium soil water content, and deep soil water content, respectively. Before running the GAM, it was necessary to check for multicollinearity issues in the model; therefore, we calculated the variance inflation factor (VIF) of the variables and removed those with a VIF > 10. After inspection of each GAM, only Ta was not eligible, so the remaining nine explanatory variables were retained in the models. Finally, to characterize the response relationships of plant phenology, we visualized each pair of response variables and explanatory variables in the model separately.

## 3. Results

### 3.1. Phenological Dynamics of Different Vegetation Types

#### 3.1.1. Scrub

**Figure 3:**
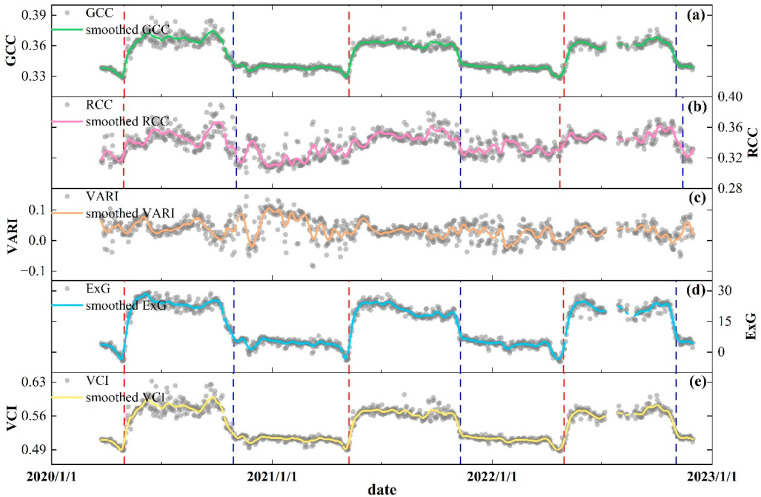
Variations of daily PhenoCam GCC (**a**), RCC (**b**), VARI (**c**), ExG (**d**), and VCI (**e**) from ROI1. The red dashed lines and blue dashed lines represent the start of season (SOS) and the end of season (EOS) for each year, respectively. SOS derived from RCC in 2021 cannot be accurately extracted. In addition, SOS and EOS also cannot be extracted in the VARI series.

Five vegetation indexes were used in ROI1 to characterize the phenological dynamics of scrub (Figure 3). Throughout the study period, GCC (Figure 3a) significantly increased from May of each year, a trend that would continue for about a month, and ExG (Figure 3d) and VCI (Figure 3e) showed the same increasing process in the spring. The change in RCC (Figure 3b) was not significant compared to GCC, but the increasing process still began in the spring. GCC, ExG, and VCI were maintained at high levels in all three summers, suggesting that scrub within ROI1 grew very vigorously at this stage. After entering the autumn, GCC gradually declined until it stabilized at lower levels in the winter; a similar process was exhibited by ExG and VCI. RCC also had a decreasing trend in the autumn of each year; however, RCC fluctuated with a greater extent in the winter period. In contrast to the other four indexes, VARI (Figure 3c) did not exhibit distinct seasonal dynamics and remained in an irregular state of fluctuation over the study period. The results in Figure 3 and Appendix A, Table A1, showed the phenological metrics extracted from the different index series. SOS and EOS derived from GCC, ExG, and VCI were almost consistent, but the length of the growing season (LOS) in 2020 was less than those in 2021 and 2022. Compared to the other two years, SOS and EOS occurred relatively late in 2021, in early May and first half of November, respectively. In addition, the RCC-derived LOS reached 187 and 204 days in 2020 and 2022, respectively, but we could not accurately extract SOS for the RCC series in 2021. VARI series cannot be extracted to phenological metrics due to irregular variations.

#### 3.1.2. Arbor Forest

**Figure 4:**
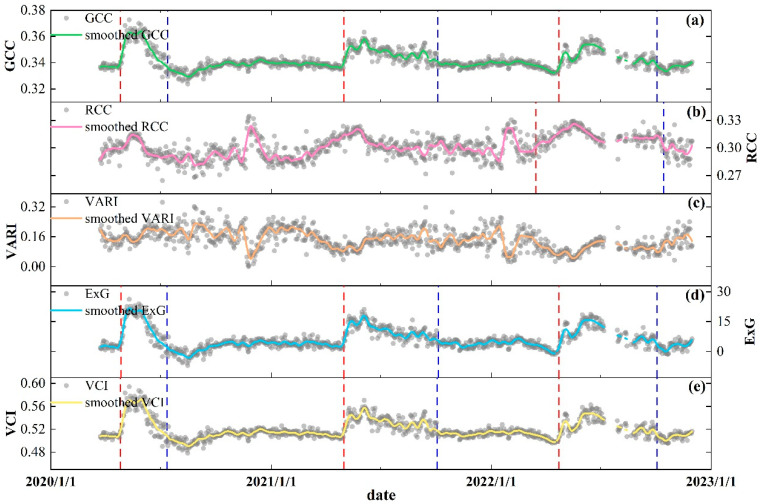
Variations of daily PhenoCam GCC (**a**), RCC (**b**), VARI (**c**), ExG (**d**), and VCI (**e**) from ROI2. The red dashed lines and blue dashed lines represent the start of season (SOS) and the end of season (EOS) for each year, respectively. For RCC and VARI, we extracted only the phenological metrics derived from RCC for the year 2022.

As shown in Figure 4, GCC (Figure 4a), ExG (Figure 4d), and VCI (Figure 4e) more consistently characterized the seasonal variations of arbor forest phenology; however, in contrast, RCC (Figure 4b) and VARI (Figure 4c) seemed to not be effective in describing such seasonal trends. During the study period, we found that GCC increased rapidly in the spring, peaked in May or June, and then gradually declined and remained at a stable level in the winter. Both ExG and VCI showed similar trends to those of GCC. EOS derived from GCC, ExG, and VCI in 2020 were significantly earlier than the other two years, resulting in the length of growing season for that year being just less than 80 days (Appendix A, Table A2). In comparison, the LOS for 2022 was 163 days as long. Although it was unable to extract phenological metrics from RCC series for the previous two years, LOS derived from RCC for 2022 exceeded LOS derived from the other three series (Appendix A, Table A2; Figure 4). For VARI, it was still unable to obtain any effective phenological metrics from these data.

#### 3.1.3. Grassland

**Figure 5:**
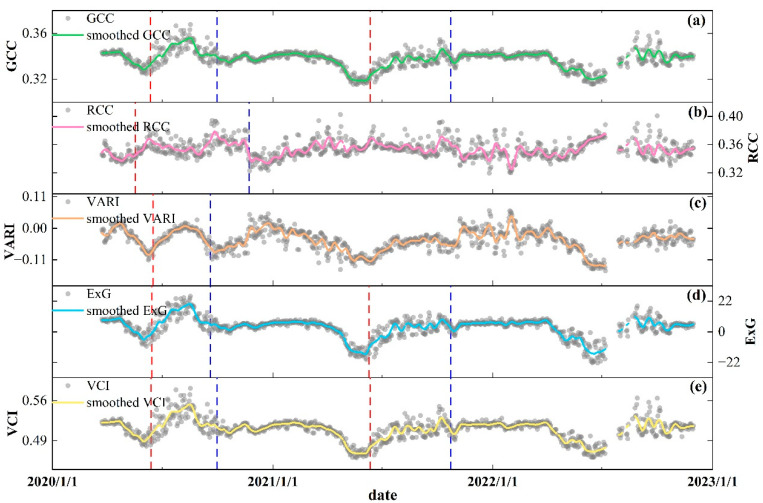
Variations of daily PhenoCam GCC (**a**), RCC (**b**), VARI (**c**), ExG (**d**), and VCI (**e**) from ROI3. The red dashed lines and blue dashed lines represent the start of season (SOS) and the end of season (EOS) for each year, respectively. For RCC and VARI, we extracted only the phenological metrics for the year 2020. Due to the lack of raw data, we were unable to obtain phenological metrics information for 2022.

Grassland phenology in 2020 had a distinct seasonal dynamic, with rapid growth almost from June until it peaked in August (Figure 5). For each index series, we extracted the phenological metrics for 2020, and the LOS derived from RCC reached the longest 189 days compared to the other four indexes (Appendix A, Table A3). The GCC, ExG, and VCI series for 2021 showed a similar change process, with LOS ranging from 133 to 136 days (Appendix A, Table A3; Figure 5). We were unable to obtain SOS information for grassland from the vegetation index series in 2022 due to missing raw observational data, but the trend suggested that grassland maintained rapid growth during the summer months (Figure 5).

### 3.2. Comparison of the Same Index in Different Vegetation Types

**Figure 6:**
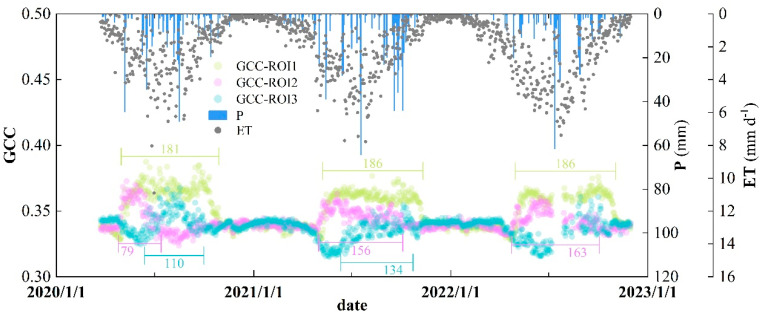
Variations of green chromatic coordinate (GCC) derived from different ROIs, as well as daily precipitation (P) and evapotranspiration (ET) processes. Different colored line segments represent the length of growing season while the numbers indicate the days.

Taking the dynamic processes of the green chromatic coordinate (GCC) extracted from three ROIs as an example, the phenological differences between various vegetation types were analyzed. As shown in Figure 6, SOS for arbor forest within ROI2 was earlier than for scrub within ROI1 and grassland within ROI3; however, EOS for scrub was later than for the other two vegetation types. SOS for grassland was later than for scrub and arbor forest, while EOS was between scrub and arbor forest. For the three vegetation types, scrub had the longest LOS, which averaged 184.3 days, and LOS for arbor forest was shorter than grassland only in 2020. From the beginning of spring each year, with the gradual increase in precipitation, the growth of vegetation was accelerated, and both scrub and arbor forest showed a clear greening process during this period. Evapotranspiration also increases during periods of vigorous vegetation growth, such as summer, with higher peaks. Therefore, these results suggested that even in a small area, each type of vegetation exhibited different phenological processes during the growing seasons and was closely related to environmental variations.

### 3.3. Relationship between GCC and Explanatory Variables

**Figure 7:**
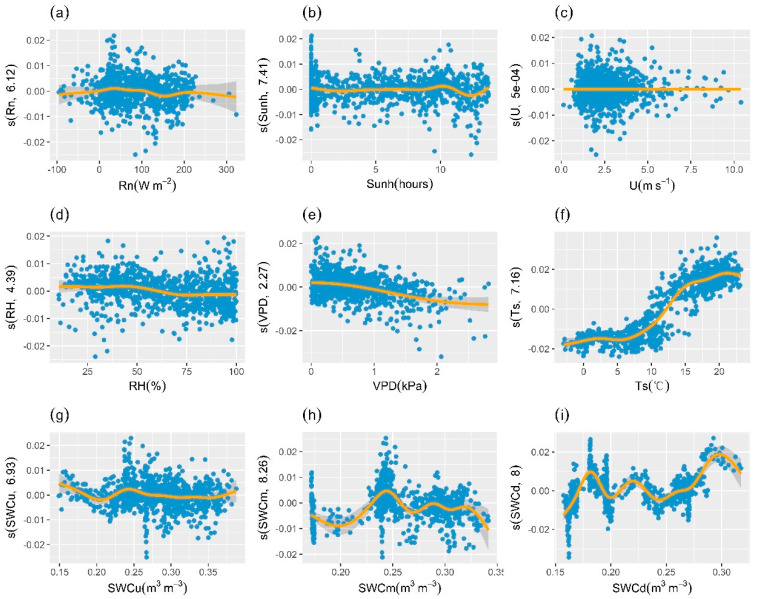
Effects of environmental factors on GCC derived from ROI1. Effect of (**a**) net radiation (Rn), (**b**) sunshine hours (Sunh), (**c**) wind speed (U), (**d**) relative humidity (RH), (**e**) vapor pressure deficit (VPD), (**f**) soil temperature (Ts), (**g**) upper soil water content (SWCu), (**h**) medium soil water content (SWCm), and (**i**) deep soil water content (SWCd) on the variation of GCC. The *y*-axis represents the partial effects of each driver. The orange line represents the smoothed fitted curve for explanatory variables. The gray shaded area represents the 95% confidence interval. The numbers in brackets in the *y*-axis labels are the effective degrees of freedom.

As shown in Figure 7, there existed nonlinear relationships between GCC series derived from ROI1 and explanatory variables, and the effects of different environmental factors on GCC differed significantly. The adjusted R^2^ of the model was 0.83 and deviance explained was 84%. The results of GAM fitting suggested that soil temperature (Ts) was the most important driver influencing GCC variation, and GCC increased rapidly with Ts between 10 °C and 15 °C. In addition, both RH and VPD had slight negative effects on GCC. In contrast, GCC was almost unaffected by wind speed (U).

**Figure 8:**
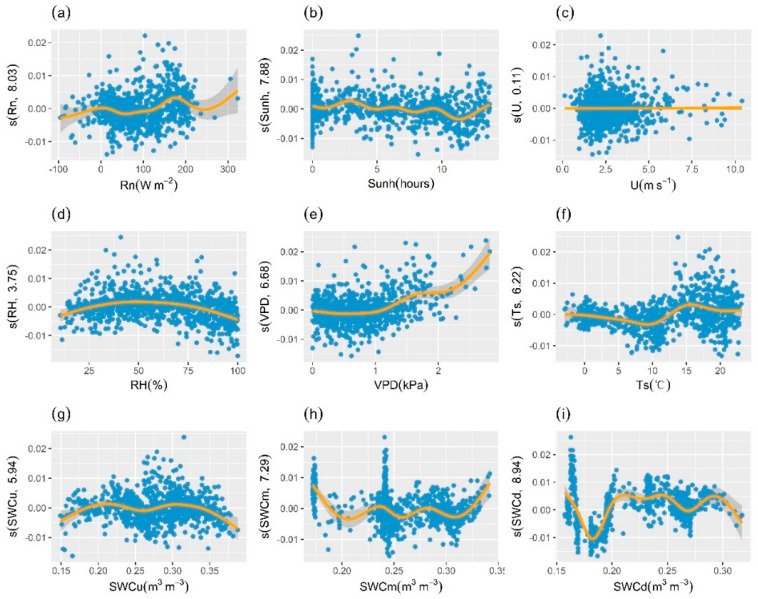
Effects of environmental factors on GCC derived from ROI2. Effect of (**a**) net radiation (Rn), (**b**) sunshine hours (Sunh), (**c**) wind speed (U), (**d**) relative humidity (RH), (**e**) vapor pressure deficit (VPD), (**f**) soil temperature (Ts), (**g**) upper soil water content (SWCu), (**h**) medium soil water content (SWCm), and (**i**) deep soil water content (SWCd) on the variation of GCC. The *y*-axis represents the partial effects of each driver. The orange line represents the smoothed fitted curve for explanatory variables. The gray shaded area represents the 95% confidence interval. The numbers in brackets in the *y*-axis labels are the effective degrees of freedom.

The results of GAM analysis showed that there was also a nonlinear relationship between GCC series derived from ROI2 and explanatory variables (Figure 8). The adjusted R^2^ of the model was 0.61 and the deviance explained was 63.3%. Deep soil water content (SWCd), vapor pressure deficit (VPD), and soil temperature (Ts) were the main factors affecting GCC. GCC as a whole increased with increasing VPD and there was a positive effect, which was obvious at high VPD. Soil temperature, as another important explanatory variable, had a negative effect with increasing Ts at less than 10 °C, after which GCC gradually increased with increasing temperature. The relationship between deep soil water content and GCC was relatively complex, and the extent of the effect differed distinctly with variation in the water content. In addition, wind speed remained without a significant effect on GCC (*p* > 0.05).

**Figure 9:**
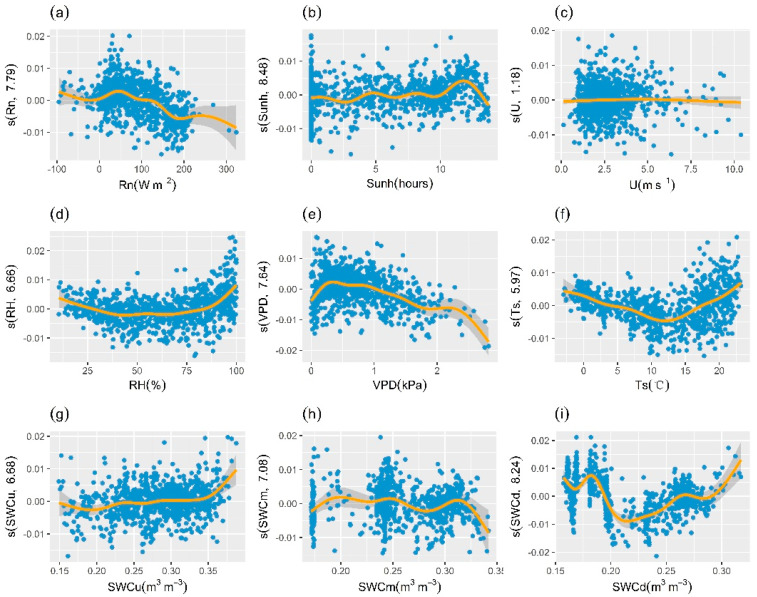
Effects of environmental factors on GCC derived from ROI3. Effect of (**a**) net radiation (Rn), (**b**) sunshine hours (Sunh), (**c**) wind speed (U), (**d**) relative humidity (RH), (**e**) vapor pressure deficit (VPD), (**f**) soil temperature (Ts), (**g**) upper soil water content (SWCu), (**h**) medium soil water content (SWCm), and (**i**) deep soil water content (SWCd) on the variation of GCC. The *y*-axis represents the partial effects of each driver. The orange line represents the smoothed fitted curve for explanatory variables. The gray shaded area represents the 95% confidence interval. The numbers in brackets in the *y*-axis labels are the effective degrees of freedom.

For the GCC series derived from ROI3, the results of the GAM analysis indicated that deep soil water content (SWCd), soil temperature (Ts), vapor pressure deficit (VPD), and relative humidity (RH) were the main impact factors. The adjusted R^2^ of the model was 0.66, and the deviance explained was 68.1%. Figure 9 shows that variation of VPD had a distinct negative effect on GCC, while GCC exhibited a decreasing and then increasing process at rising RH. Soil temperature was another important explanatory variable, which had a positive effect on GCC after about 10 °C. The effect of SWCd on GCC followed a nonlinear trend, which was positive at higher water content. In addition, the effect of wind speed was still insignificant (*p* > 0.05).

## 4. Discussion

In this study, we continuously monitored the vegetation phenology characteristics in a gully area of the Loess Plateau using PhenoCam, then explained the relationships between environmental factors and response variables based on a generalized additive model (GAM). Over a period of about two and a half years of observation, PhenoCam captured the seasonal variations in the phenology of different types of vegetation. Previous studies based on satellite remote sensing have suggested that the SOS of vegetation on the Loess Plateau was mainly concentrated on days of 96 to 144 [51], for which we have extracted SOS in scrub and arbor forest that were roughly in agreement with this result, but slightly later in grassland. In the deciduous broad-leaved forest region of the Loess Plateau, SOS was mainly distributed from days of 103 to 150 [52], which was also a consistent result. The EOS of the Loess Plateau was roughly at October [51,53]. In contrast, we found that there was delayed EOS of the scrub in the study area, occurring between the end of October and beginning of November (Table A1). In this study, there were significant differences in the phenological metrics obtained from the time series originating from ROI2, and the possible reasons might be the premature decrease in chlorophyll content of vegetation in ROI2 for 2020, which resulted in the canopy becoming darker in color and more rapidly changing in greenness. Of course, the accumulation of more observational data in the future could explain these reasons more deeply.

On the other hand, we compared the differences between different vegetation indexes in describing the phenological variations and discuss the possible reasons. For different types of vegetation, the phenological metrics from GCC, ExG, and VCI series were in close agreement. The consistency of phenological metrics extracted from GCC and VCI data was demonstrated in the United States study [40], where such indexes from the same sensor with spectral similarities had consistent trends in phenoperiod prediction [54]. In contrast, we found that RCC and VARI were not performing well, even though both were also applied to track canopy phenology [41,55]. An unmanned aerial vehicle (UAV)-based phenology monitoring, also on the Loess Plateau, suggested that GCC has more potential compared to RCC for estimating autumn canopy phenodates in hilly areas [56]. VARI was linearly related to the relative content of anthocyanins in plant leaves [57]. Anthocyanin concentration increased during leaf senescence, generally in response to biotic and abiotic stresses [41]. However, in our study, VARI showed irregular change processes when representing vegetation phenology, which performed less well than GCC, ExG, and VCI. There is still no uniform conclusion on the optimal vegetation index for monitoring phenological changes at the canopy scale, and GCC, RCC, and ExG have been used more frequently in many studies [58]. GCC is insensitive to light conditions, which reduces the effect of light sensitivity on the image, while in the meantime, GCC can well capture the change of greenness during vegetation growth; GCC also performed well in this study. Weil, et al. [59] suggested that ExG and GCC were highly correlated in the study describing phenological changes based on different vegetation indexes, and both were sufficiently sensitive to changes in greenness. In addition, the VCI, as a nonlinear transformation of the GCC, inherently provides a wider dynamic range than the GCC and contributes to the extraction of phenological metrics [16]. Compared to the other indexes, RCC and VARI did not seem to perform better, possibly because these indexes are better suited to reflect the characteristics of fall foliage discoloration, such as the process of decreasing greenness and red pigment accumulation [41]. For vegetation that experiences significant foliage discoloration in the fall, the RCC will capture these changes well. However, vegetation in this study area did not show a distinct reddening process during the decline, which resulted in inferior performance of RCC and VARI. Overall, it needs further investigation regarding the potential of different vegetation indexes in tracking phenological variations.

Environmental factors play an important role in regulating seasonal variations of ecosystem phenology, which typically include temperature, photoperiod, and soil moisture [8,60,61,62]. In this paper, our results found that soil temperature was a significant factor that influenced GCC from different types of plants in the study region, especially for scrub phenology. When spring snow was melted by solar radiation, some of the snow water flowed into soil and promoted root activity, while soil temperature directly affected root growth and uptake of water and nutrients by roots [63]. Therefore, soil temperature regulated plant growth as well as the microbial functions of soil [63,64]. In addition, autumn phenology may also be affected by soil temperature [65]. In general, we suggested that soil temperature was the important driver, but it was not mean to be the only factor, such as soil water content and vapor pressure deficit could also influence the plant phenology, as evidenced by previous studies [66,67,68]. Therefore, it is obvious that the driving mechanisms behind vegetation phenological changes are complex and varied, and further investigation into the possible interactions between different factors controlling phenology is necessary.

## 5. Conclusions

The study utilized near-surface remote sensing (PhenoCam in this paper) methods to monitor phenological changes in different types of vegetation. We set three ROIs on each image to represent different plants, including scrub, arbor forest, and grassland, and then calculated the vegetation index derived from each ROI. Finally, we analyzed the environmental factors that affected plant phenology in the study region.

GCC, ExG, and VCI were all able to characterize the phenology of scrub, arbor forest, and grassland well, compared to RCC and VARI, which did not performed well. The SOS for scrub occurred from late April to early May, while the EOS appeared from late October to early November, and LOS derived from GCC ranged between 181 and 186 days. For arbor forest, SOS also occurred in late April through early May, except in 2020, and EOS was in early October. Almost all of SOS in grassland were in early June, while EOS was distributed in September and October.

The SOS of arbor forest was earlier than that of scrub and grassland, while EOS of scrub was later than that of the other two vegetation types. Scrub had the longest LOS, which averaged 184.3 days. With increasing precipitation, both scrub and arbor forest showed a greening process. The evapotranspiration also peaked during periods of vigorous plant growth.

There were nonlinear relationships between GCC series and explanatory variables. The R^2^ of the GAM fitting was the highest in ROI1 and was 0.83. The results indicated that soil temperature was an important environmental factor that influenced GCC changes. For different vegetation types, these drivers also included deep soil water content, vapor pressure deficit, and relative humidity. In contrast, the effect of wind speed on GCC was not significant.

There are a few experiments on long-term monitoring of vegetation based on PhenoCam at a small scale on the Chinese Loess Plateau, and our work explored the phenological processes of different plants in the same environment. Therefore, these results can help to improve our understanding of vegetation phenology on the Loess Plateau and provide references for related studies.

## Figures and Tables

**Table 1:** Vegetation indices calculated from red (R), green (G), and blue (B) digital numbers in PhenoCam images.

Index	Equations	References
Green chromatic coordinate	GCC = G/(R + G + B)	[42]
Red chromatic coordinate	RCC = R/(R + G + B)	[41]
Visible atmospherically resistant vegetation index	VARI = (G − R)/(R + G − B)	[41]
Excess green index	ExG = 2G − (R + B)	[22]
Vegetation contrast index	VCI = G/(R + B)	[40]

## Data Availability

The experimental data used in this study were obtained from the Chunhua Ecohydrology Experimental Station of Xi’an University of Technology and the dataset is named Chunhua Ecohydrology Experimental Station Dataset (CEESD), which can be obtained from the corresponding author (Dengfeng Liu, liudf@xaut.edu.cn) upon personal reasonable request.

## Appendix A

Table A1 lists the start of season (SOS), end of season (EOS), and length of growing season (LOS) information extracted from the GCC, RCC, ExG, and VCI series. It should be noted that these information in Table A1 were derived from ROI1.

**Table A1:** List of SOS, EOS, and LOS for each year in ROI1.

Index	Year	SOS	EOS	LOS (Days)
GCC	2020	30 April	28 October	181
	2021	8 May	10 November	186
	2022	30 April	2 November	186
RCC	2020	29 April	2 November	187
	2021	-	9 November	-
	2022	23 April	13 November	204
ExG	2020	30 April	29 October	182
	2021	8 May	9 November	185
	2022	29 April	2 November	187
VCI	2020	30 April	28 October	181
	2021	8 May	9 November	185
	2022	30 April	2 November	186

Table A2 lists the start of season (SOS), end of season (EOS), and length of growing season (LOS) information extracted from the GCC, RCC, ExG, and VCI series. It should be noted that these information in Table A2 were derived from ROI2.

**Table A2:** List of SOS, EOS, and LOS for each year in ROI2.

Index	Year	SOS	EOS	LOS (Days)
GCC	2020	25 April	13 July	79
	2021	1 May	4 October	156
	2022	23 April	3 October	163
RCC	2020	-	-	-
	2021	-	-	-
	2022	16 March	14 October	212
ExG	2020	26 April	12 July	77
	2021	1 May	5 October	157
	2022	23 April	3 October	163
VCI	2020	25 April	12 July	78
	2021	1 May	3 October	155
	2022	23 April	3 October	163

Table A3 lists the start of season (SOS), end of season (EOS), and length of growing season (LOS) information extracted from the GCC, RCC, VARI, ExG, and VCI series. It should be noted that these information in Table A3 were derived from ROI3.

**Table A3:** List of SOS, EOS, and LOS for each year in ROI3.

Index	Year	SOS	EOS	LOS (Days)
GCC	2020	12 June	30 September	110
	2021	11 June	23 October	134
	2022	-	-	-
RCC	2020	17 May	22 November	189
	2021	-	-	-
	2022	-	-	-
VARI	2020	16 June	19 September	95
	2021	-	-	-
	2022	-	-	-
ExG	2020	14 June	19 September	97
	2021	9 June	23 October	136
	2022	-	-	-
VCI	2020	13 June	30 September	109
	2021	12 June	23 October	133
	2022	-	-	-
